# Screening of immunosuppressive cells from colorectal adenocarcinoma and identification of prognostic markers

**DOI:** 10.1042/BSR20203496

**Published:** 2021-04-06

**Authors:** Fazhan Li, Jun Zhou, Zedong Li, Leiyi Zhang

**Affiliations:** 1 Zhengzhou University, The Fifth Affiliated Hospital of Zhengzhou University, Zhengzhou, Henan, China; 2 Central South University, Xiangya Second Hospital, Changsha, Hunan, China

**Keywords:** COAD, Immunosuppressive cells, Th-17 cells

## Abstract

Background: Colorectal cancer (CRC) is the most common type of gastrointestinal malignant tumour. Colorectal adenocarcinoma (COAD) – the most common type of CRC – is particularly dangerous. The role of the immune system in the development of tumour-associated inflammation and cancer has received increasing attention recently.

Methods: In the present study, we compiled the expression profiles of 262 patients with complete follow-up data from The Cancer Genome Atlas (TCGA) database as an experimental group and selected 65 samples from the Gene Expression Omnibus (GEO) dataset (of which 46 samples were with M0) as a verification group. First, we screened the immune T helper 17 (Th17) cells related to the prognosis of COAD. Subsequently, we identified Th17 cells-related hub genes by utilising Weighted Gene Co-expression Network Analysis (WGCNA) and Least Absolute Shrinkage and Selector Operation (LASSO) regression analysis. Six genes associated with the prognosis in patients with COAD were identified, including: *KRT23, ULBP2, ASRGL1, SERPINA1, SCIN*, and *SLC28A2*. We constructed a clinical prediction model and analysed its predictive power.

Results: The identified hub genes are involved in developing many diseases and closely linked to digestive disorders. Our results suggested that the hub genes could influence the prognosis of COAD by regulating Th17 cells’ infiltration.

Conclusions: These newly discovered hub genes contribute to clarifying the mechanisms of COAD development and metastasis. Given that they promote COAD development, they may become new therapeutic targets and biomarkers of COAD.

## Background

Colorectal cancer (CRC) is the most common type of gastrointestinal malignancy. It is the third most commonly diagnosed cancer globally and the fourth leading cause of cancer-related mortality [1]. Colorectal adenocarcinoma (COAD) is the most common type of CRC, with more than a million new cases and more than 600000 deaths worldwide each year [2,3]. Risk factors for COAD include genetic mutations, environmental factors such as smoking and obesity, and various inflammatory diseases [4–6]. So far, surgical treatment is still the most commonly used treatment. Although it has prolonged patients’ survival time to a certain extent, the 5-year survival rate in patients with metastases is still below 10% [7]. Therefore, it is essential to further unravel the pathogenesis of COAD and explore new treatment options.

Lymphocyte infiltration has been detected in animal and human tumours for more than a century [8]. In the 1970s, Hamlin first reported the relationship between lymphocyte infiltration and tumour prognosis in breast cancer patients [9]. It has been confirmed that the degree of tumour immune infiltration positively correlates with tumour non-metastasis and that 80% of tumour-infiltrating cells are T cells [10]. For example, CD8^+^ T cells are associated with the prognosis of colorectal tumours [11]. In recent years, immunotherapy has made significant progress for advanced cancer, such as melanoma and non-small cell lung cancer [12,13]. T helper 17 (Th17) cells are a subset of T cells, which differentiate from CD4^+^ T cells, secrete interleukin (IL) 17 (IL-17), and play an essential role in autoimmune diseases and defence responses [14]. Th17 cells cause tissue damage and organ damage by secreting proinflammatory cytokine IL-17 [15]. Th17 cells play an essential role in the pathogenesis of diseases, such as gastric cancer [16,17], ovarian cancer [18], non-small cell lung cancer [19], and breast cancer [20]. Recent studies using the CRC murine model have shown that up-regulation of IL-17 signalling promotes tumour growth and progression. Th17 cells and IL-17 play a direct role in tumour-associated inflammation and cancer development [21,22]. Interestingly, subsequent studies indicated that Th17 cells in the epithelium were significantly associated with patient survival, suggesting that Th17 cells have a dual role in CRC [23]. This result caught our attention, and we verified and further explored it in this experiment.

In the present study, we used data from the The Cancer Genome Atlas (TCGA) and Gene Expression Omnibus (GEO) datasets to identify genes associated with Th17 cells and constructing Weighted Gene Co-expression Network Analysis (WGCNA) and Least Absolute Shrinkage and Selector Operation (LASSO) regression analysis. WGCNA is a method to analyse gene expression patterns in multiple samples. Genes with similar expression patterns are clustered, and the correlation between the modules and specific traits or phenotypes can be calculated. In the present study, WGCNA-based methods were used to identify gene modules correlated with colon cancer development, and the most representative genes were identified as hub genes to help in screening for colon cancer. A risk scoring model was constructed based on the finally determined hub genes to evaluate the prognosis of COAD. The close relationship between these hub genes and Th17 cells may provide new ideas for COAD treatment.

## Methods

### Data collection

The expression profiles and clinical data of patients with COAD were obtained from the Cancer Genome Atlas (TCGA) cohort and the Gene Expression Integrated Library (GEO). The present study included the expression profiles of 262 patients with complete follow-up data in the TCGA database (Histological type: COAD; M0 in TNM stage, which means that there were no distant metastases), and from the GEO dataset, 65 samples were taken (of which 46 were with M0). The TCGA biolinks package and the GEO query package were used to download TCGA and GEO data [24,25]. All data and sample information in the present study were extracted from the databases and did not require the ethics committee review.

### Research method

**Figure 1:**
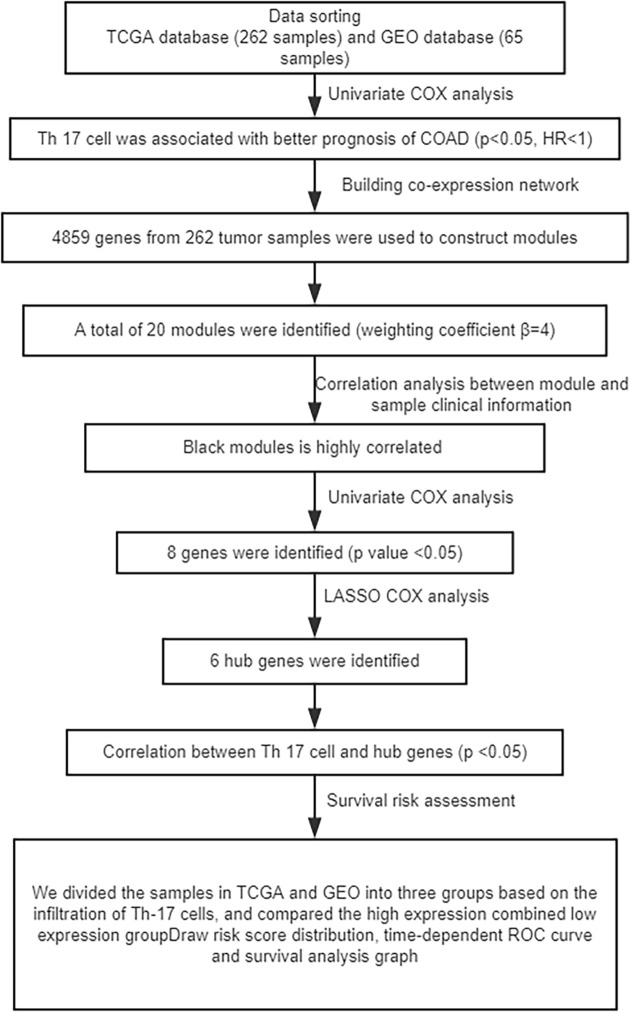
Flow chart

The overall study design and the different samples that were included at every stage of the analysis are illustrated as a flowchart in Figure 1.

### Infiltration of immune cells

ssGSEA was performed on TCGA and GEO gene expression profile data to analyse the infiltration of immune cells in tumour tissues quantitatively. In total, 24 immune cell types were infiltrated. ssGSEA enrichment score calculated the extent to which genes in a particular gene set in a single sample were coordinated up-regulated or down-regulated. ssGSEA ranked genes by their absolute expression in the sample and calculated enrichment scores by integrating the differences between empirical cumulative distribution functions at the gene level [26,27].

### Univariate Cox regression analysis and Kaplan–Meier’s curve

We screened for immune cells that may affect COAD by univariate Cox analysis in the TCGA and GEO datasets (*P*<0.05). We used the ‘survminer’ package for optimal separation statistics, divided gene expression into high-expression and low-expression groups, and plotted Kaplan–Meier curves.

### Construction of co-expression network and identification of related modules

The co-expression network was constructed from the WGCNA package in R [28]. We first selected genes with variances more significant than all variance quartiles in the TCGA dataset (Top quarter, because those genes with larger variances mean more significant variation in different samples). Then, we identified the expression data for the selected genes, and clustered the samples to detect outliers. Finally, the gene clustering module was determined based on clinical features and TOM-based differences [29]. The correlation between module characteristic genes (MEs) and clinical features was calculated to identify highly relevant gene clustering modules.

### Hub gene determination and risk scoring model construction

In the present study, we selected modules positively correlated with disease characteristics (screening based on residual tumour, pathological stage, OS, OS events, and Th17 cells, where positive and negative values indicated a positive and negative correlation, respectively, while the absolute value of *P*-value denoted the magnitude of correlation). We used univariate Cox analysis to screen for genes that were significantly associated (*P*<0.05) with prognosis in the module, and finally LASSO. The screening of COAD prognosis-related genes based on lambda.min (lambda corresponding to the minimum mean error) was selected for the hub genes; LASSO was analysed using the glmnet package in R. Central gene expression values weighted by LASSO regression coefficients yielded a risk score for each patient. The Survminer R software package was used to find the best cut-off value for the risk score. The ROC and Kaplan–Meier curve were used to assess the predictive ability of the risk score.

## Results

### Quantifying immune cell infiltration and analysis of the relationship between immune cells and tumour recurrence

**Figure 2:**
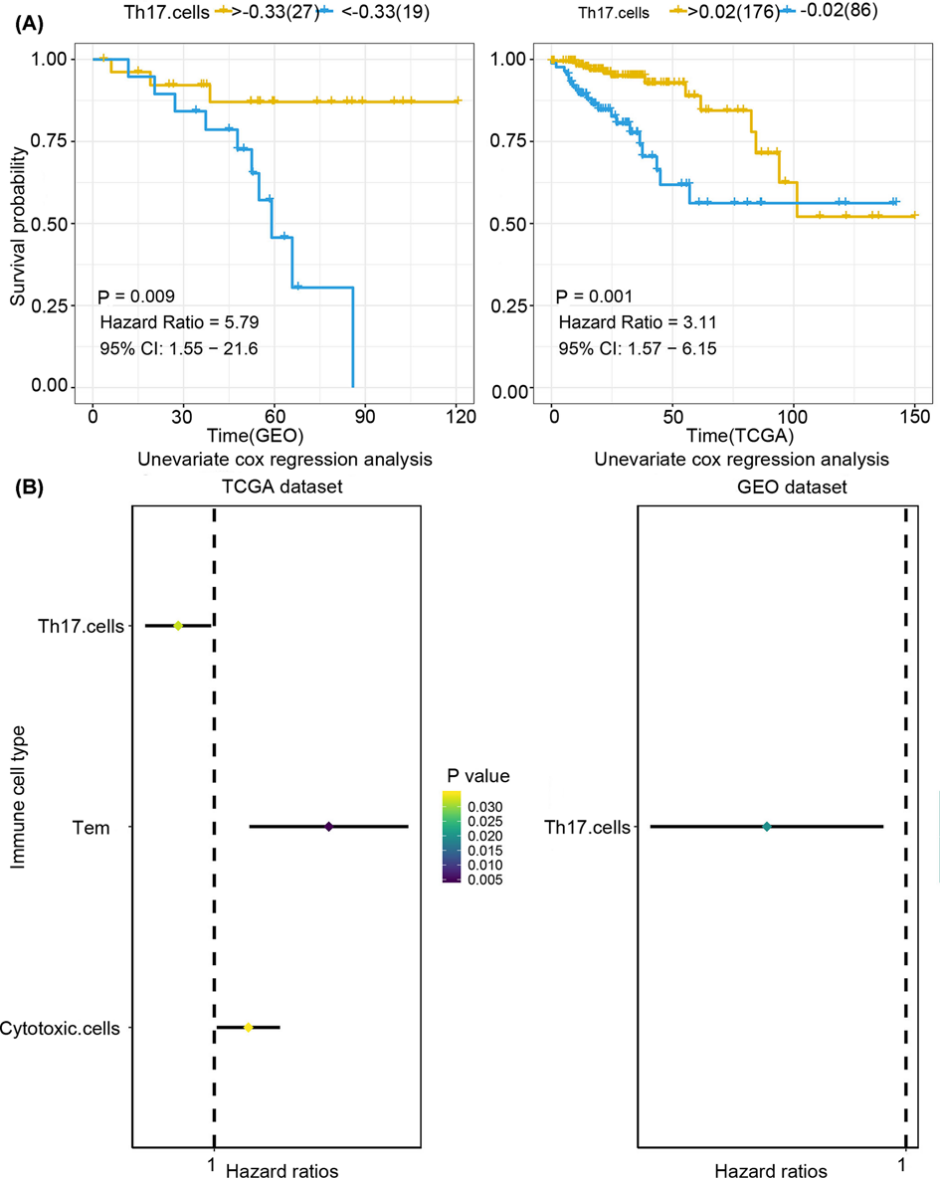
Quantifying immune cell infiltration in TCGA and GEO samples (**A**) Kaplan–Meier curves of Th17 cells in immune cells. Genetic analysis of the TCGA and GEO databases showed that the high-invasion group had a significant inhibitory effect on the prognosis of COAD, and Th17 cells were associated with a better prognosis (GEO samples: *P*=0.009, HR = 5.79, 95% CI: 1.55−21.6, TCGA samples: *P*=0.001, HR = 3.11, 95% CI: 1.57−6.15). (**B**) Forrest plot of univariate Cox regression analysis in COAD (Th17 cells were associated with prognosis and HR < 1, both as protective factors in both TCGA and GEO).

First, we used single-sample gene set enrichment analysis (ssGSEA) to quantify mRNA data from 25 immune cell infiltrations in TCGA and GEO samples, namely, aDC, B cells, CD8^+^ T cells, cytotoxic cells, DCs, eosinophils, iDCs, macrophages, mast cells, neutrophils, NK CD56 bright cells, NK CD56dim cells, NK cells, pDC, T cells, T helper cells, Tcm, Tem, TFH, TFH, Tgd, Th1 cells, Th17 cells, Th2 cells, and Tregs. Immune cells associated with COAD tumours were then screened by Cox univariate analysis. We found that Th17 cells had significant association (*P*<0.05) with COAD both in TCGA and GEO datasets, with Hazard Rate (HR) below 1 (Figure 2A), which indicated that Th17 cells play a protective role in COAD. The overall study design and the different samples that were included at every stage of the analysis are illustrated as a flowchart in Figure 1.

The ‘survival’ and ‘survminer’ R packages were used to determine the relationship between Th17 cells and COAD prognosis, and a Kaplan–Meier curve was plotted (Figure 2B). We found that higher degree of infiltration of Th17 cells was associated with a better prognosis of COAD (GEO samples: *P*=0.009, HR = 5.79, 95% CI: 1.55−21.6; TCGA samples: *P*=0.001, HR = 3.11, 95% CI: 1.57−6.15).

### Construction of the co-expression network and identification of related modules

**Figure 3:**
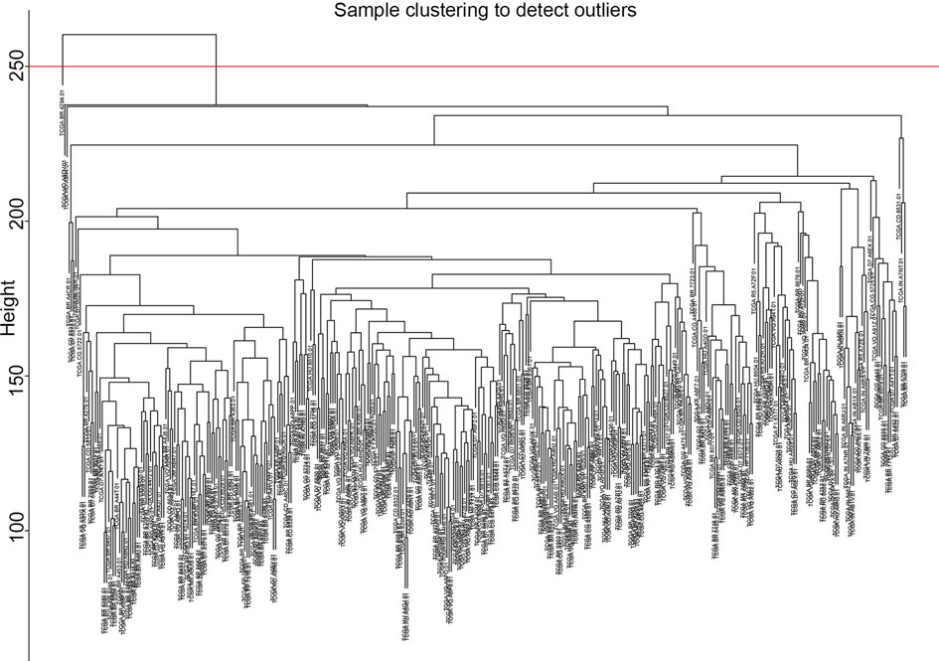
Systematic clustering of 262 COAD tumour samples and clinical information

**Figure 4:**
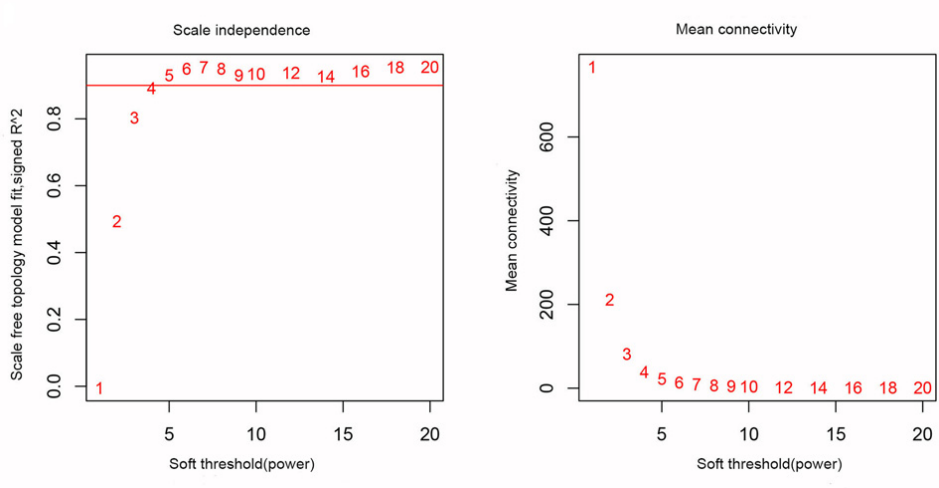
Scale-free conformance index and average connectivity calculated at different β values (the numbers in the figure represent the corresponding soft threshold power. An approximate scale-free topology can be achieved at a soft threshold power of 4)

**Figure 5:**
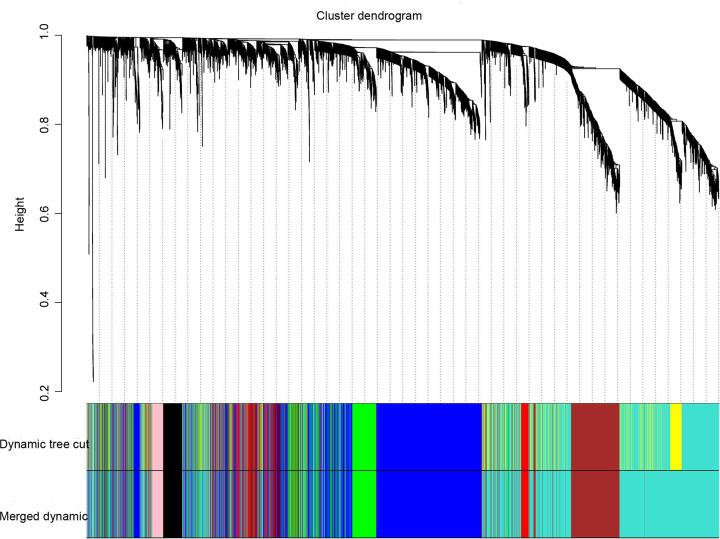
Gene-cluster tree diagram Based on consensus topological overlap, each colour module represents a colour-coding module containing a set of highly connected genes. Each module includes at least 50 genes (larger modules are relatively more meaningful).

In the present study, we calculated the first quarter of the variance for each gene expression in all samples to construct a co-expression network with a total of 4859 genes. A hierarchical clustering tree was then built for 4859 genes out of 262 samples, and 12 outlier samples were eliminated (Figure 3). A hierarchical clustering tree was constructed for the remaining 250 samples. We chose the weighting coefficient β value of 4 to build the co-expression network while moderately retaining each gene node (Node) (Figure 4). The similar modules were then combined by a hybrid dynamic shear tree method to identify 20 modules and colour representation of each sample’s phenotype (white for low, red for high, and grey for missing; Figure 5).

### Correlation between modules and cancer

**Figure 6:**
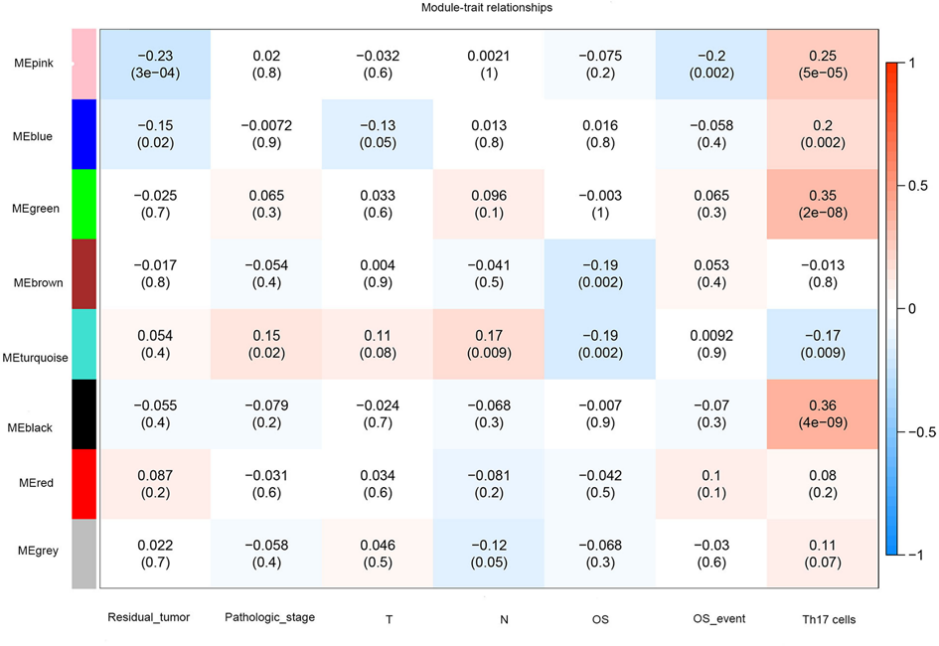
Heat map of different modules with different clinical features Each row corresponds to a consistency module, and each column corresponds to one type of clinical information (screening based on the residual tumour, pathological stage, overall survival (OS), OS events, and Th17 cells). The absolute value of the *P*-value indicates the correlation size (the module name is displayed on the left side of each cell, and the associated intensity and direction are shown on the right side of the heat map).

We selected a significant correlation module by comparing the clinical information of each module in COAD patients. Among them, the black module (cor = 0.36, *P*=4 × 10^−9^) showed the highest correlation with Th17 cells (positive values indicating positive correlation and negative values indicating negative correlation), demonstrating that Th17 cells may affect the prognosis of COAD patients by regulating the genes of the black module (Figure 6).

### Identification of hub genes

**Figure 7:**
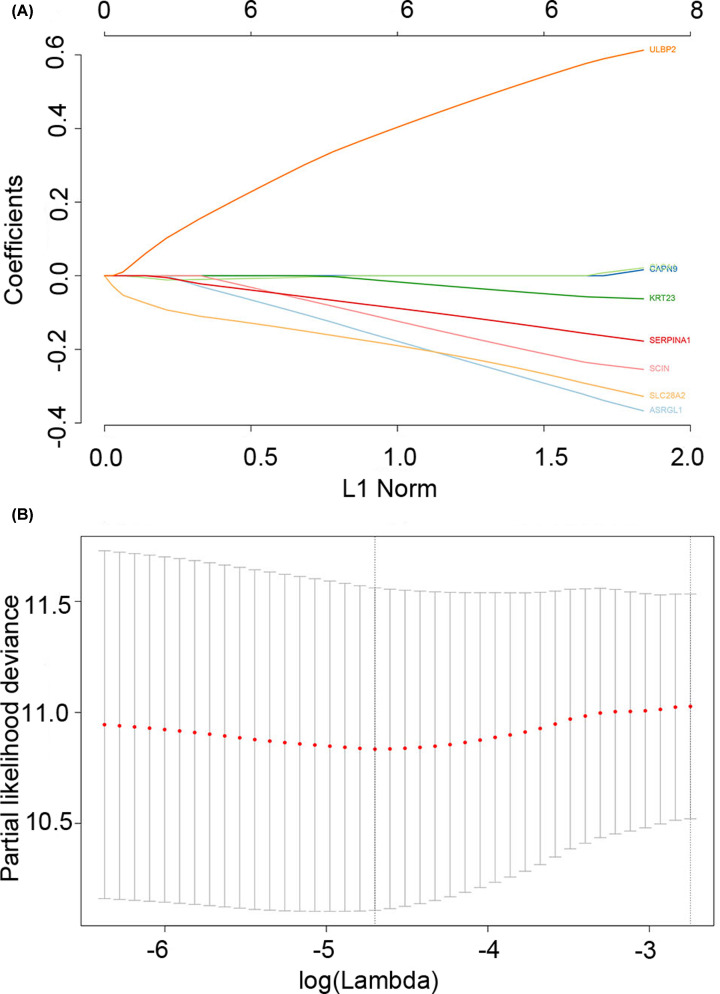
LASSO regression analysis (**A**) Distribution of LASSO coefficients for eight related genes. (**B**) Partial likelihood bias of the LASSO coefficient distribution. The vertical dashed line indicates the minimum partial likelihood deviation.

The black module had 207 genes, of which 160 genes were identical with the GEO dataset. To further identify the genes associated with COAD, we performed a Cox univariate analysis on the same 160 genes in both datasets, setting the *P*-value threshold at 0.05. Eight related prognostic genes were screened. Then, LASSO regression analysis was used to screen eight prognosis-related genes in the black module (the best λ value was 0.009100874, and the more concise model λ value in the SE was 0.06420486) (Figure 7). It was finally determined that six genes (*KRT23, ULBP2, ASRGL1, SERPINA1, SCIN*, and *SLC28A2*) were related to patients’ prognosis. We used LASSO regression analysis and univariate Cox analysis to screen for related prognostic genes. The identified hub genes can be considered to be related to the prognosis given that *P*-values were below 0.05.

### Risk score

**Figure 8:**
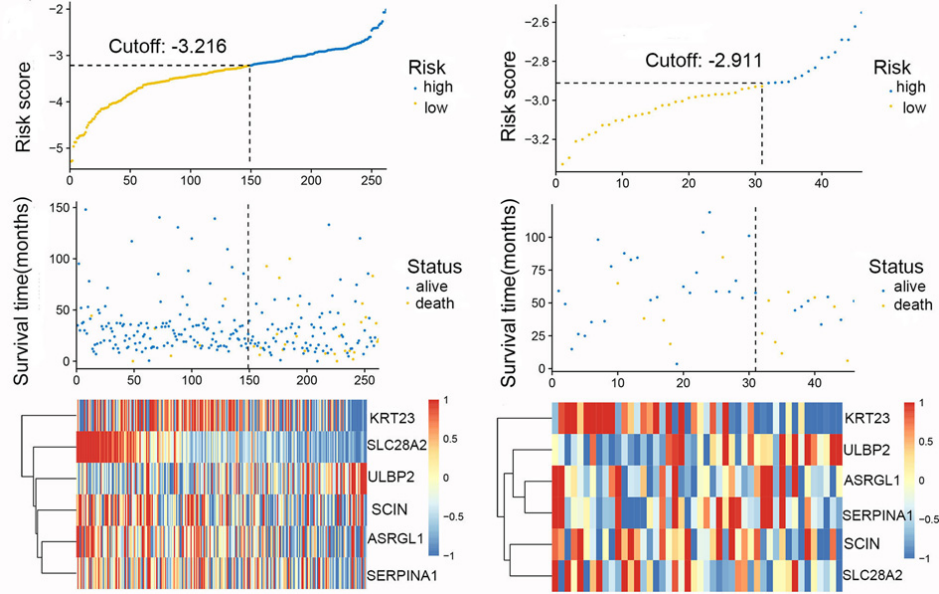
Relationship between risk scores and the expression levels of the six hub genes

**Figure 9:**
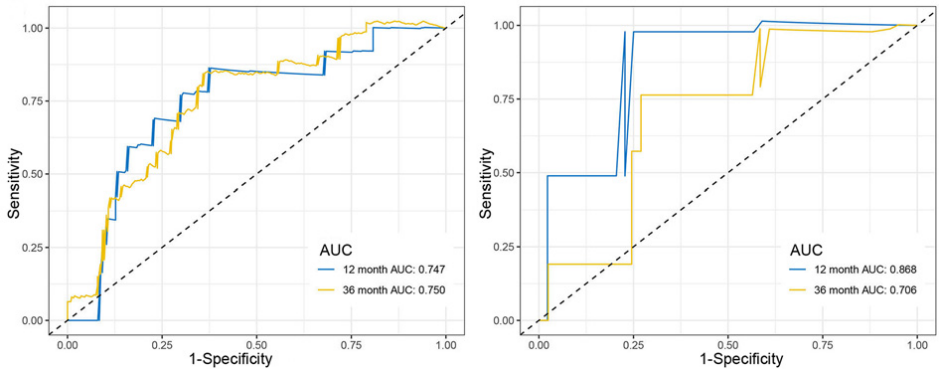
Time-dependent ROC curve analysis for clinical prediction models

**Figure 10:**
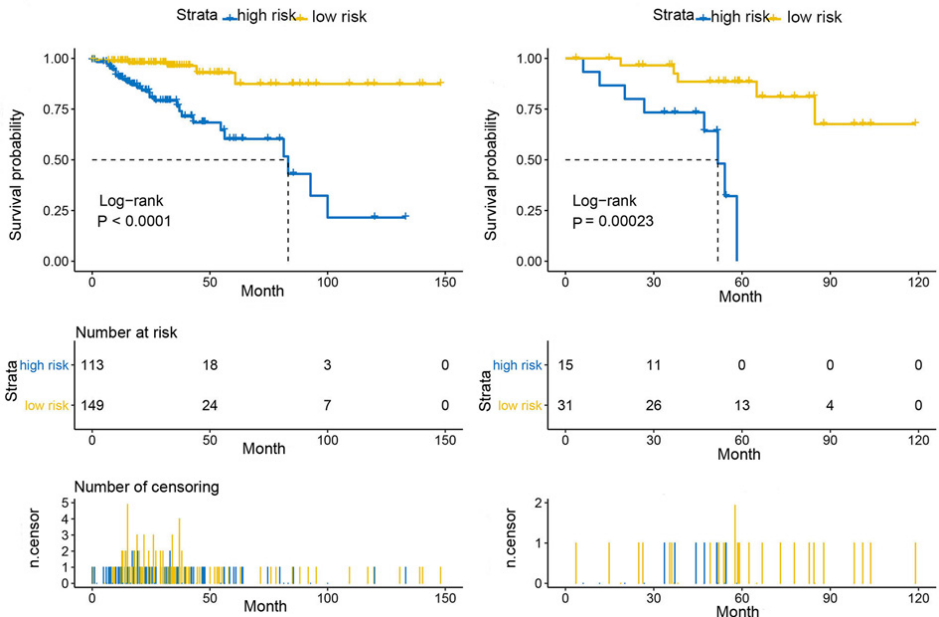
Relationship between high- and low-risk scores and OS The OS of the low-risk score group was significantly higher compared with the high-risk score group.

The optimal cut-off for the risk scores was found by Survminer R package, while Receiver Operating Characteristic (ROC) and Kaplan–Meier curve were used to assess the predictive capacity of the risk scores. We first divided the samples from TCGA and GEO datasets into three groups based on the infiltration of the Th17 cells, compared the high-expression with low-expression group, and plotted the risk score distribution (Figure 8), time-dependent ROC curve (Figure 9), and survival analysis of the TCGA and GEO datasets (Figure 10). The area under the ROC curve (AUC) of the OS prognostic model at 12 months was 0.747 (TCGA) or 0.868 (GEO), while at 36 months AUC was 0.750 (TCGA) or 0.706 (GEO).

## Discussion

In the present study, we first evaluated the amount of Th17 cells in COAD patients. We found that Th17 cells were significantly reduced in COAD patients, suggesting that Th17 cells are closely related to COAD. Th17 cells and their IL-17 have been considered to play an essential role in developing many types of malignancies [19,20,30–35]. IL-17 promotes tumour growth through various mechanisms, including inhibiting specific immune cell infiltration in hepatocellular carcinoma and melanoma [36,37]. The extracellular matrix can remodel IL-17 to regulate the surrounding microenvironment and promote the invasion and metastasis of cancer cells [38–40]. Recent mouse models of CRC have shown that Th17 cells and IL-17 have a direct role in tumour-associated inflammation and cancer development [22].

However, the dual role of Th17 cells in CRC should be taken seriously. The conclusions from our experiment further validated the protective role of Th17 cells in the prognosis of CRC patients and identified the relevant hub genes. These results contribute to the study of immunologically-relevant targeted therapies for COAD.

Although the role of IL-17 in the mechanism of tumorigenesis has been confirmed, there have been relatively few studies to further determine its related genes. In the present study, we identified the relevant genes ‘KRT23 ULBP2 ASRGL1 SERPINA1 SCIN SLC28A2’ that may affect Th17 cells’ immune infiltration in COAD patients. These genes are involved in the development of many diseases [41–48]. In non-gastrointestinal tumours, ASRGL1 has shown a high correlation with the prognosis of patients with locally advanced lymph node-negative prostate cancer and cervical cancer [41,49]. SLC28A2 helps increase the role of ribavirin (RBV) in the treatment of viral hepatitis Hyperuricemia (HUA) has been implicated [47,50]. KRT23 was identified as a specifically expressed and highly expressed gene in CRC [44]. Comprehensive analysis of the lncRNA–miRNA–mRNA network also further confirmed that ULBP2 was associated with the prognosis of CRC [51]. In the serine protease inhibitor protein family (SERPIN), serpinA1 as a biomarker in CRC can increase the predictive ability of CRC diagnosis [52], and it has been shown that Snail and serpinA1 promote CRC progression through fibronectin. In this context, serpinA1 may be a potential therapeutic target for novel predictive biomarkers and CRC. Scinderin (SCIN) may be a significant predictor of poor prognosis in patients with CRC liver metastasis (CRLM) and CRC [53]. Our research provided new insights into the genes highly expressed in CRC, which may help explain the dual role of Th17 cells.

The results of the present study further confirmed the role of these six genes in COAD. These results also confirmed the protective effects of these six genes in COAD, and indicated that the effects of these genes on Th17 cells may be the cause of their dual products. It also means that ‘KRT23 ULBP2 ASRGL1 SERPINA1 SCIN SLC28A2’ may be considered a potential target and biomarker for treatment. Although we used clinical samples and verified them with advanced analysis methods and multiple databases, some limitations of our research should be mentioned. On one hand, the expression levels of the genes we identified were not determined through further experiments. Besides, the number of samples also had certain limitations. On the other hand, the relationship between the hub gene and Th17 was based on target prediction. Further molecular biology experiments are needed to prove the interaction.

## Conclusions

In conclusion, we compiled the expression profiles of 262 patients with complete follow-up data from the TCGA database as an experimental group and selected 65 samples from the GEO dataset. Next, we identified the Th17 cells-related hub genes by constructing co-expression network analysis (WGCNA) and lasso regression analysis (LASSO). Our results indicated that the genes associated with Th17 cells (‘KRT23 ULBP2 ASRGL1 SERPINA1 SCIN SLC28A2’) may play a significant role in COAD in humans. The difference in Th17 cells between the M0 samples and samples with metastases may be related to the above-mentioned six genes. Our findings may improve our understanding of the mechanism of tumour seeding and metastasis in COAD, but further studies are needed to validate our results.

## Data Availability

The data used in the present study were derived from TCGA and GEO databases.

## Abbreviations

AUC: area under the receiver operating characteristic curve
COAD: colorectal adenocarcinoma
CRC: colorectal cancer
DC: Dendritic Cells
GEO: Gene Expression Omnibus
IL: interleukin
LASSO: Least Absolute Shrinkage and Selector Operation
NK: natural killer cell
ROC: receiver operating characteristic
SCIN: Scinderin
ssGSEA: single-sample gene set enrichment analysis
TCGA: The Cancer Genome Atlas
Th17: T helper 17
WGCNA: Weighted Gene Co-Expression Network Analysis
